# Development and validation of the General Rehabilitation Adherence Scale (GRAS) in patients attending physical therapy clinics for musculoskeletal disorders

**DOI:** 10.1186/s12891-020-3078-y

**Published:** 2020-02-01

**Authors:** Atta Abbas Naqvi, Mohamed Azmi Hassali, Syed Baqir Shyum Naqvi, Sadia Shakeel, Madiha Zia, Mustajab Fatima, Wajiha Iffat, Irfanullah Khan, Amnah Jahangir, Muhammad Nehal Nadir

**Affiliations:** 1 Discipline of Social and Administrative Pharmacy, School of Pharmaceutical Sciences, Universiti Sains Malaysia, 11800 Penang, Malaysia; 2 Faculty of Pharmacy, Hamdard University, Karachi, 74400 Pakistan; 3 Dow College of Pharmacy, Dow University of Health Sciences, Karachi, 75270 Pakistan; 4 Institute of Physical Medicine and Rehabilitation, Dow University of Health Sciences, Karachi, 75270 Pakistan; 5 Discipline of Clinical Pharmacy, School of Pharmaceutical Sciences, Universiti Sains Malaysia, 11800 Penang, Malaysia; 6 Department of Pharmacy, Ziauddin University Hospital, Karachi, 74700 Pakistan

**Keywords:** Physical therapy specialty, Musculoskeletal diseases, Validation studies, Questionnaire designs, Treatment adherence and compliance

## Abstract

**Background:**

Non-adherence to physical therapy ranges from 14 to 70%. This could adversely affect physical functioning and requires careful monitoring. Studies that describe designing and validation of adherence measuring scales are scant. There is a growing need to formulate adherence measures for this population. The aim was to develop and validate a novel tool named as the General Rehabilitation Adherence Scale (GRAS) to measure adherence to physical therapy treatment in Pakistani patients attending rehabilitation clinics for musculoskeletal disorders.

**Methods:**

A month-long study was conducted in patients attending physical therapy sessions at clinics in two tertiary care hospitals in Karachi, Pakistan. It was done using block randomization technique. Sample size was calculated based on item-to-respondent ratio of 1:20. The GRAS was developed and validated using content validity, factor analyses, known group validity, and sensitivity analysis. Receiver operator curve analysis was used to determine cut-off value. Reliability and internal consistency were measured using test-retest method. Data was analyzed through IBM SPSS version 23. The study was ethically approved (IRB-NOV:15).

**Results:**

A total of 300 responses were gathered. The response rate was 92%. The final version of GRAS contained 8 items and had a content validity index of 0.89. Sampling adequacy was satisfactory, (KMO 0.7, Bartlett’s test *p*-value< 0.01). Exploratory factor analysis revealed a 3-factor model that was fixed and confirmed at a 2-factor model. Incremental fit indices, i.e., normed fit index, comparative fit index and Tucker Lewis index, were reported > 0.95 while absolute fit index of root mean square of error of approximation was < 0.03. These values indicated a good model fit. The value for Cronbach (α) was 0.63 while it was 0.77 for McDonald’s (ω), i.e., acceptable. Test-retest reliability coefficient was 0.88, *p* < 0.01. Education level was observed to affect adherence (*p* < 0.01). A cut-off value of 12 was identified. The sensitivity and accuracy of the scale was 95%, and its specificity was 91%.

**Conclusion:**

The scale was validated in this study with satisfactory results. The availability of this tool would enhance monitoring for adherence as well as help clinicians and therapists address potential areas that may act as determinants of non-adherence.

## Introduction

The Global Burden of Disease 2013 Report estimates that worldwide life expectancy has improved to about 6 years and healthy life expectancy about 5.4 years. Besides, roughly an eighth of life expectancy was linked to living with disability. This reduction in global mortality has shifted the epidemiological paradigm from communicable to non-communicable diseases [1]. According to the World Report on Disability, more than one billion people are affected by some form of disability globally and out of which 200 million have severe functional difficulties owing to their disability. The non-communicable diseases especially the musculoskeletal illnesses also contribute to the increasing number of disabled patients. The 2015 Millennium Development Goals stress on the need of empowering people with disabilities and improving their quality of life as well as physical functioning [2].

Physical therapy (PT) is a type of specialty care given by physiotherapists that aims to reduce the disability related pain, improves functional capacity and adjust the disability in life thereby improving the patients’ quality of life [3–5]. Adhering to the physical therapy schedule may improve a patient’s health outcomes and achieve the goals of therapy [5]. A systematic review by Peiris and colleagues reported that physical therapy improves activity and participation outcomes as well as reduce hospital stay. It further highlighted that physical therapy may be cost-effective [6]. For instance, physical therapy advised to patients with disabilities resulted from cerebrovascular illness such as stroke as well as from musculoskeletal illnesses such as rheumatoid arthritis, osteoarthritis and osteoporosis, may improve patient’s functional capacity [7]. Studies report that 15–100 min of physical activity in a day reduces 4% likelihood of mortality [5, 8]. Hence, it is imperative to follow the instructions and rehabilitation schedule as advised by physiotherapists.

Evidence indicates that patients may tend to forego appointments and may not adhere to their prescribed PT schedule. Adherence to PT remains undocumented at large [9]. Studies in the past have estimated that non-adherence to physical therapy may range from 14 to 70% [5, 9–11]. These finding highlight the existence of several barriers to rehabilitative service. These barriers may either be patient oriented or related to organization. Jack and colleagues mentioned that researches have mostly focused on patient related barriers to rehabilitation services [9]. There is a need to investigate other determinants such as economic and logistical issues that may prove to be obstacles. Naqvi and colleagues reported that patients undergoing physical therapy mentioned financial constraints and unavailability of physiotherapists, as barriers alongside treatment resulted pain [3].

The concept of physical therapy adherence is multidimensional. According to Kolt and colleagues, adherence may encompass treatment attendance, concordance to PT’s advice and, undertaking of prescribed exercises [9, 12, 13]. Hence, there is a need to document a patient’s physical therapy adherence to evaluate the treatment outcomes as well as identify potential barriers. This is not only beneficial for the patients but would also contribute positively to the quality assessment and improvement of health services.

Several studies have endeavored to measure adherence to exercise however, only few had used questionnaires as an outcome measure. In a systematic review, Uzawa and Davis reported that eight studies were conducted during 1996–2015 that measured adherence to a home-based exercise program [14]. However, only three studies utilized questionnaires while others used a patient-reported diary to document frequency of clinic visits, as a measure of adherence. Of the three studies that used a questionnaire, Hartigan and colleagues used the visual analogue scale (VAS) and Owestry disability index to measure pain and physical functioning as proxy for compliance/adherence [15]. Murray and colleagues used the Treatment Self-Regulation Questionnaire to measure motivation of patients towards their exercise program as a proxy for adherence [16]. Besides, Medina-Mirapeix and colleagues used a Likert based single item questionnaire for measuring adherence that was originally developed by Sluijs and colleagues in 1993 [17, 18]. Another study conducted in Turkey measured adherence to home-based exercise on a five-point ordinal scale [19]. Most importantly none of these measures were validated in this population. This approach of measuring adherence by a variety of unvalidated instruments and indirect methods highlights an undeniable need to formulate and validate a dedicated tool that specifically measures patient’s adherence to physical therapy and exercise.

Pakistan is a developing country in South Asia with a population over 200 million [20]. Data from Institute for Health Metrics and Evaluation (IHME) reports that back pain and musculoskeletal disorders are the most common causes for disability while stroke is one of the most common causes for death and disability combined [21]. According to the Pakistan Bureau of Statistics (PBS) 2012 Report, the country has a disability rate of 2.65% and, there are over 5 million physically disabled persons in Pakistan [20]. Much of the advancement in the field of PT has been made in the last decade. Physical therapy education was a 4-year bachelor program until 2008 when it was revised to a 5-year Doctor of Physical Therapy (DPT) degree. The first association for the profession of physical therapy was formed in 2008 and first collaboration with international regulatory body was established in 2011. The formation of the first ever PT regulatory body is currently in process [20, 22]. According to the World Confederation for Physical Therapy (WCPT) 2017 Report, it is estimated that there are 15,000 registered PT working in Pakistan. There are between 1 and 5 registered therapists per 10,000 patients in Pakistan [23].

Several determinants have been identified that hinder Pakistani patients in attending physical therapy sessions. Naqvi and colleagues highlighted that exhaustive treatment duration, treatment attendance, treatment resulted pain, delayed results and, out-of-pocket expenditure, were notable issues that acted as determinants of non-adherence to PT in Pakistan [3, 18]. Patients may not understand the importance of therapy and hence may not prioritize it over other commitments [24]. Besides, in a qualitative study, patients highlighted that unavailability of a female therapist often act as a determinant of non-adherence. It may sometimes cause inconvenience as female patients may refuse treatment by a male therapist, considering religious and societal norms [3]. Moreover, patients highlighted accessibility issues such as unavailability of caregivers who could accompany patients to clinics, as a reason for not attending PT sessions [18]. Furthermore, it was reported that physical therapist offering discounts to patients who could not afford the cost of PT sessions have helped increase patient retention [3].

Considering limitations of available tools as well as determinants of non-adherence in a developing country like Pakistan, we aimed to develop and validate a novel tool termed as the General Rehabilitation Adherence Scale (GRAS) to measure adherence to physical therapy, rehabilitation and exercise in Pakistani patients with musculoskeletal disorders.

## Methods

A study was conducted for a month (March 2018) in the Department of Physical Therapy at Clifton Hospital and Institute of Physical Medicine and Rehabilitation, Dow University Hospital, Karachi Pakistan.

### Study aim

The aim of the study was to develop and validate a novel self-reporting scale named as the ‘General Rehabilitation Adherence Scale’, in Urdu language to measure adherence to physical therapy, rehabilitation and exercise in Pakistani patients with musculoskeletal disorders.

### Recruitment and randomization

The study used the block randomization technique. The data collection was conducted daily from 9 am to 1 pm in the day and from 3 pm to 9:30 pm in the evening. We used the patients’ appointment numbers to randomize the sample. Patients with an even-numbered appointment were invited to participate in the study and odd-numbered appointments were left out. The sequence was reversed the other day, i.e., odd-numbered appointments were invited. A computer-generated list was used for this purpose. This approached helped in eradication of bias in selection.

### Participants and eligibility criteria

Patients who had to undergo physical therapy for any musculoskeletal condition for at least 2 weeks were invited to participate in the study. The study included both male and female patients who were adult and above 18 years. Patients were asked to provide their written consent before handing the questionnaire. Those patients who were not willing to participate were left out. Incomplete questionnaires were not included in the study.

### Sampling technique and sample size

We conducted random sampling and the sample size was calculation was based on item response theory, i.e., item-to-respondent ratio. The questionnaire contained eight items and therefore an item-to-respondent ratio of 1:20 was considered enough for validation purpose. The required sample size was calculated by the following formula:

$$ N={n}_i\ x\ R $$

Where, N = sample size; n_i_ = number of items in questionnaire and R = item-to-respondent ratio. The sample size obtained was 160. A drop-out rate of 10% which was roughly 20, was added to yield final sample size of 180 patients.

### Research instrument development and conceptualization

A novel research tool named as the General Rehabilitation Adherence Scale (GRAS) was formulated and used for this study. Prior to instrument development, a thorough literature review was carried out [3, 7]. The tool was developed in Urdu language since it was the local language of Pakistanis. The initial draft of the tool contained 13 items related to adherence regarding physiotherapy. All items were multiple choice questions (MCQs) and were graded. The draft was subjected to review by a panel of experts and was validated with eight items.

### Face and content validity

The initial draft was subjected to face and content validity. A panel of experts was formed that comprised of two pharmacists, two physiotherapists, a rheumatologist, an occupational therapist, two academicians and a social scientist. The panel carried out face and content validity of GRAS. In addition, patients were also consulted in the process. Face validation was conducted through Delphi consensus method [25]. The content validity index (CVI) and ratio (CVR) were calculated by asking every expert to mark each item in the questionnaire as essential or non-essential, from the perspective of a patient [26, 27]. The content validity ratio was calculated using the following formula:

$$ CVRi=\frac{n\raisebox{1ex}{$e-N$}\!\left/ \!\raisebox{-1ex}{$2$}\right.}{\raisebox{1ex}{$N$}\!\left/ \!\raisebox{-1ex}{$2$}\right.} $$

Where, *CVRi* is the content validity ratio of each item; *n*^*e*^ was the number of experts indicating essentiality of item and *N* is the total number of experts in the panel. Content validity index (CVI) was analysed by calculating mean ratio of the tool using the following formula.

$$ CVI=\left[ CVR1+ CVR2+ CVR3+\dots CVR13\right]/13 $$

### Determination of cut-off values

Receiver operating curve (ROC) analysis was used to determine cut-off value for the scale [28]. The focus of ROC analysis was to determine a score that had highest sensitivity and lowest inverse of specificity [29]. The scoring of GRAS was later defined based on cut-off values.

### Patient adherence levels and scoring criteria

Based on cut-off value, we defined adherence to rehabilitation as patient’s concordance to the physical therapy schedule and categorized it into five levels, i.e., high, good, partial, low and poor. The scoring criterion was defined along with the levels of adherence. The final content validated draft of the GRAS had a total of eight items that awarded a maximum score of 24. Each item of the tool had four possible options namely, always, mostly, sometimes and never, that awarded an individual score of 0, 1, 2 and 3 respectively. A patient would be categorized as being ‘highly’ adherent if his score is between 20 and 24. Similarly, patients would be classified as being ‘good’ in adherence if the final score is between 17 and 19 and ‘partial’ if the score is between 12 and 16. A patient would be considered having a ‘low’ adherence if the score is between 8 and 11 and ‘poor’ if the score is 7 or less. The Urdu and English versions of GRAS and its scoring code are available as Additional files 1 and 2.

### Factor analyses

The factor structure of GRAS was examined through Exploratory factor analysis (EFA) using Principle component analysis (PCA) with Varimax rotation. It was carried out with an item-to-respondent ratio of 1:20. The model was then confirmed through Partial confirmatory factor analysis (PCFA) using Maximum likelihood analysis with same rotation. The incremental fit indices namely the comparative fit index (CFI), normed fit index (NFI) and Tucker Lewis index (TLI) were calculated. Additionally, absolute fit index, i.e., root mean square error of approximation (RMSEA) was reported [30, 31].

### Internal consistency and reliability analyses

The internal consistency was calculated by test-retest method using Cronbach’s alpha (α) values. The value was considered satisfactory if it was ≥0.5. Besides, intra-class correlation (ICC) was also calculated [5, 30]. Alternatively, the McDonald’s coefficient (ω_*t*_) was also calculated as an estimate of reliability [32, 33]. The test-retest reliability between two time-points was assessed after a gap of 4 weeks through Pearson’s correlation coefficient (ρ). A value of (ρ) more than 0.75 and *p*-value < 0.05 was considered significantly strong correlation [30, 31].

### Known group validation

We believed that educated patients would be more adherent to their prescribed PT schedule as compared to their uneducated counterparts. The known group validity was evaluated through chi square (χ^2^) test and a p-value less than 0.05 was considered acceptable.

### Sensitivity analysis

Sensitivity analysis was conducted to determine the GRAS’s sensitivity to identify adherent patients correctly, as well as its specificity, i.e., to accurately distinguish non-adherent patients. Sensitivity, specificity, accuracy and predictive values were reported as a percentage (%) and in 95% confidence interval range. Likelihood ratios were reported as a value and in 95% confidence interval range. Standard logit confidence interval method was used to report predictive values [28, 34]. Log method was used for determination of likelihood ratios and Clopper-Pearson method was used to calculate sensitivity, specificity and accuracy of the scale [35].

### Questionnaire administration

The GRAS questionnaire was handed to patients after obtaining their consent. Patients filled in their responses returned the questionnaire. Apart from GRAS, the patients were also provided with a demographic questionnaire that contained questions related to age, gender, education, occupation, income, residence, health insurance and comorbidity. A second copy of the scale was filled by patients at 2nd timepoint. The three forms, i.e., demographic questionnaire and, GRAS at 1st and 2nd timepoints were kept together in a separate file designated for a patient using his/her medical record number as identifier.

### Data analysis

All data were analyzed through IBM SPSS version 22, Armonk, NY, USA. The demographic data was reported in sample counts (N) and percentages (%). The associations between demographic and adherence variables were tested by chi square (χ^2^) test for association. Statistical significance was considered at *p*-value less than 0.05.

### Ethics approval and patient consent

The patients were explained about the objectives of the study and their written consent was sought. The study was approved by the Institutional Review Board of Allied Med Ethics (Ref# NOV:15) and Department of Physiotherapy, Clifton Hospital Karachi.

## Results

### Face and content validity

The initial draft of the scale consisted of 13 items. This draft was subjected to a panel of experts. Three items were modified. The minimum content validity ratio (CVR) required to retain an item was 0.78. A total of 5 items were dropped based on low CVR. Apart from a low ratio, all experts agreed that items 3, 4 and 13 of the original draft were unnecessary and believed that items 1 and 2 had already covered items 3 and 4, while item 13 made no sense. Besides, items 7 and 8 were considered repetitive. The original GRAS with face and content validity results is available as Additional file 3. The content validity index (CVI) was 0.89 after dropping 5 items, and the scale was finalized with 8 items. The final version of GRAS with scoring code is available as Additional file 2. The CVI and CVR are reported in Table 1.

**Table 1:** Content validity ratio and factor structure

GRAS items	Items content	CVR	Component	
			1	2
1	Other commitments	0.80	0.717	
2	Unable to manage time	0.84	0.794	
3	Feel well	0.94	0.746	
4	Excessive pain	0.87	0.794	
5	Treatment cost	0.99		0.668
6	Not worth the money spent	0.8		0.609
7	Unavailability of caregiver	0.99		0.627
8	Unavailability of therapist	0.96		0.524

*CVR* content validity ratio

### Pilot results of the instrument

A total of 326 patients were enrolled in the study after providing their consent out of which 300 patients returned filled questionnaires twice. The response rate was 92%. The average age of patients was 44 ± 14.5 years. Most patients were females (*N* = 184, 61.3%), had basic education (*N* = 225, 75%), were married (*N* = 254, 84.7%), lived in urban localities (*N* = 243, 81%) and were associated with household activities (*N* = 135, 45%). Most patients had no medical insurance (*N* = 268, 89.3%), no comorbidity (*N* = 182, 62%) and a monthly family income between PKR 25,000 – 50,000, i.e., USD 188.1–376.3. The mean adherence score was reported at 15 ± 4.7, while the median was 16. Lowest score obtained was 4 and highest was 24. A third of patients had partial adherence (*N* = 100, 33.3%). The value of USD corresponds to the USD to PKR exchange rate at the time of this writing, i.e., 1st Nov 2018. The demographic information is tabulated in Table 2.

**Table 2:** Participants information

Participants information (*N* = 300)	N	%
Gender		
Male	116	38.7
Female	184	61.3
Education		
Primary education	225	75
Uneducated	75	25
Marital status		
Single	46	15.3
Married	254	84.7
Occupation		
Employed	99	33
Unemployed	33	11
Retired	11	3.7
Household	135	45
Self-employed	22	7.3
Monthly Family Income		
Less than PKR 10,000 (<USD 75.3)	73	24.3
Between PKR 10,000–25,000 (USD 75.3–188.1)	99	33
Between PKR 25,000–50,000 (USD 188.1–376.3)	82	27.3
more than PKR 50,000 (>USD 376.3)	46	15.3
Residence		
Urban	243	81
Rural	57	19
Health insurance		
Full insurance	4	1.3
Partial insurance	28	9.3
No insurance	268	89.3
Comorbidity		
Yes	114	38
No comorbidity	186	62
GRAS adherence score interpretation		
High Adherence = 20–24 points	51	17
Good Adherence = 17–19 points	82	27.3
Partial Adherence = 12–16 points	100	33.3
Low Adherence = 8–11 points	47	15.7
Poor Adherence = 0–7 points	20	6.7

1 USD equals 132 PKR

### Factor analyses

The factor structure of GRAS was analyzed through EFA using PCA with Varimax rotation. The Kaiser-Mayer-Olkin (KMO) measure of sampling adequacy was reported at 0.7 with significant Bartlett’s test of sphericity, i.e., *p*-value< 0.01. A 3-factor solution was obtained with eigenvalues > 1.0, that accounted for 62.3% of variance. Factor 1 constituted 28.4% of variance while factor 2 and 3 contributed 19.9 and 14% of variance respectively. Items with factor loading > 0.5 on a component and, non-salient loading < 0.5 on another component, were considered as a single factor. This demonstrated a clear factor structure. As a result of the scree plot and the secondary factor analysis, factors 2 and 3 were combined to measure factors related to accessibility. Factor 1 concerned individual patient factors (Table 1). The secondary factor analysis provided a 2-factor solution that had better factor loadings and internal consistency.

**Fig. 1:**
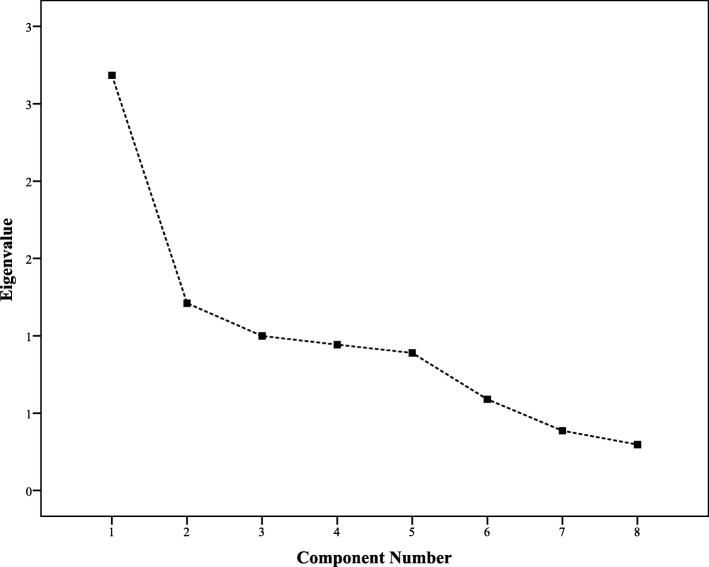
Scree plot

The 2-factor model was then confirmed through PCFA using MLA with oblimin rotation (Fig. 1). The value of KMO was reported at 0.693 with significant Bartlett’s test of sphericity, i.e., *p*-value< 0.01. The non-salient factor loading distribution curve was normal with mean value of 0.3. The null-model χ2 was reported at 499.375 while the implied model χ2 was reported at 16.378. The values obtained in PCFA for NFI was 0.97, 0.99 for CFI and 0.98 for TLI. All values were greater than 0.95, The value for RMSEA was 0.02 which was less than 0.03. The values indicated a good model fit.

### Internal consistency and reliability analysis

**Fig. 2:**
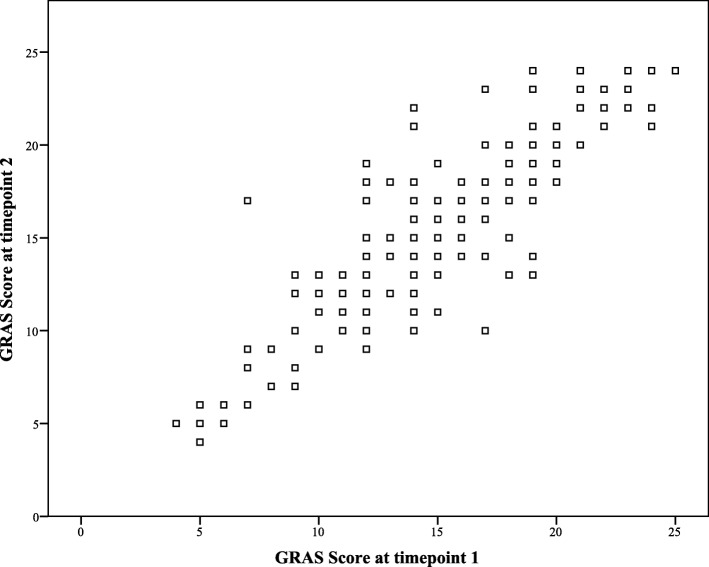
Test-retest correlation of GRAS scores at timepoints 1 and 2

The overall reliability of GRAS for eight items was reported a Cronbach alpha (α) value of 0.63, i.e., acceptable with intraclass correlation of 0.63 (0.56–0.69). The McDonald’s coefficient (ω_*t*_) was reported at 0.77, i.e., satisfactory. The reliability of component 1 was reported at 0.623 with ICC range of 0.485–0.712 for 95% CI. The component 2 had an alpha value of 0.764, ICC = 0.709–0.809 for 95% CI. The test-retest reliability of GRAS was assessed by correlating the rehabilitation adherence scores of participants at timepoints 1 and 2. The test-retest correlation coefficient was reported at 0.88 (*p*-value< 0.01) (Fig. 2).

### Known group validity

It was evaluated by cross tabulating the adherence score with demographic variable of education. We found that most patients who had basic education appeared adherent as compared to uneducated patients (*p*-value< 0.001) (Table 3).

**Table 3:** Cross tabulation between patients’ education and adherence

Educational Status		GRAS Score interpretation for adherence				
		High	Good	Partial	Low	Poor
Educated	Count (expected)	45 (38.3)	51 (61.5)	71 (75)	39 (35.3)	19 (15)
	% within Educational status	20	22.7	31.6	17.3	8.4
Uneducated	Count (expected)	6 (12.8)	31 (20.5)	29 (25)	8 (11.8)	1 (5)
	% within Educational status	8.0%	41.3	38.7	10.7	1.3

### Determination of cut-off value

**Fig. 3:**
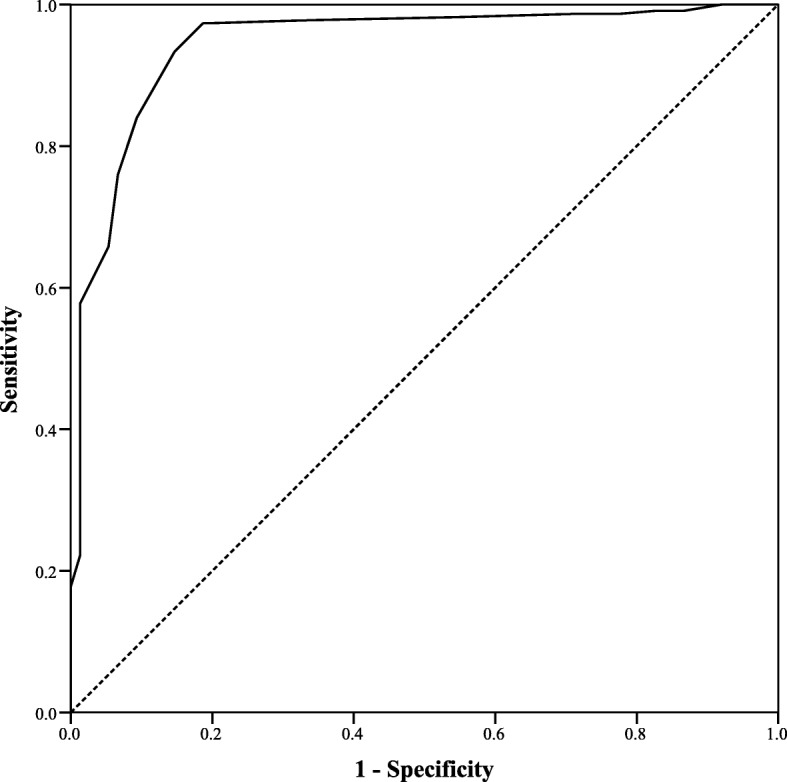
Receiver operating curve

The ROC analysis highlighted 223 positive states while 77 were negative. A positive state meant that the patient was adherent. The ROC calculated an area under the curve (AuC) of 94.5%, i.e., 0.945 ± 0.16 (0.913–0.976 for 95% CI) (*p*-value< 0.001). Based on the coordinates of ROC analysis, a cut-off value of 12 was selected to distinguish adherent patients from non-adherent ones. The sensitivity of GRAS at score of 12 was 93.3% while inverse of specificity was 10.3%. The ROC is available as Fig. 3.

### Sensitivity analysis

The sensitivity of GRAS was 95.8% (93.05–97.68% for 95% CI) and its specificity was 91.04% (81.52–96.64% for 95% CI). The (+) likelihood ratio was 10.7 (4.98–22.96 for 95% CI) while (−) likelihood ratio was 0.05 (0.03–0.08 for 95% CI). The positive predictive value was 98.15% (96.12–99.13% for 95% CI) and negative predictive value was 81.33% (72.18–87.97% for 95% CI). The accuracy of GRAS was reported at 95% (92.38–96.92% for 95% CI).

## Discussion

This study was conducted to develop and validate a novel tool to document adherence to physical therapy, rehabilitation and exercise in patients attending physical therapy sessions for musculoskeletal illnesses. The tool addressed multiple domains that acted as determinants to adherence in this population. Crombie and colleagues reported that despite the belief that physical activity is beneficial, most of the respondents either had no interest in attending physical therapy sessions or faced accessibility issues. Some patients also suffered from physical symptoms such as shortness of breath and pain [36]. Rodrigues et al. mentions that adherence to physical therapy is difficult and may result from combination of various determinants [37]. The impact of out-of-pocket cost for treatment as a determinant to adherence in Pakistani patients attending physical therapy sessions was recently investigated [3, 18, 38, 39]. A qualitative study in Pakistani poliomyelitis survivors revealed that direct cost of treatment was perceived as a barrier to poliomyelitis rehabilitation treatment [3]. Moreover, another study highlighted that a significant number of poliomyelitis patients who had low income routinely forego their treatment in face of a financial crisis [18]. The GRAS not only addressed patient-related and logistical factors but cost-related non-adherence as well. Since, it was developed for Pakistani patients therefore, it was in Urdu language.

Our scale reported a similar CVI to the one reported by Rodrigues and colleagues for their scale that measured determinants to treatment among osteoporosis treatment [37]. However, it was primarily developed for osteoporosis patients and its generalizability was a limitation. Moreover, the length of the questionnaire and the time required to fill it was also regarded as a limitation. The GRAS contained eight items and was developed primarily with a holistic approach to measure adherence in patients who require physical therapy for various musculoskeletal illnesses. The validity of sports injury rehabilitation scale developed by Kolt and colleagues was analyzed using EFA only [12]. However, the validation of GRAS included EFA as well as PCFA. Hence, measurement purification of GRAS was more stringent. We also calculated McDonald’s coefficient (ω) as an alternate reliability while previous tools estimated reliability using Cronbach (α) values only. These psychometric features distinguish GRAS from other scales.

Apart from these aspects, the scale also established a satisfactory test-retest validity that demonstrated the stability of the scale over time. One of the most notable features of this scale was the determination of a cut-off value for designating patients as adherent and non-adherent. This was carried out using receiver operator curve (ROC) analysis. This aspect was absent in validation process of previous tools. Besides, using a Likert-based approach to differentiate between adherent and non-adherent patients used by Chan and colleagues may not be as sensitive as ROC analysis [19]. The original scale developed by Sluijs and colleagues designated patients as adherent or non-adherent, based on assumptions. Sluijs and colleagues acknowledged that reliability of their scale could not be estimated [11].

Most studies used patient diaries and alternate questionnaires that measured another outcome as a proxy for adherence. Such measures do not qualify as benchmark to judge effectiveness of GRAS. Only three questionnaires are available in literature that measured adherence to exercise directly. In comparison with those questionnaires [11, 14, 15, 17], GRAS is novel in the sense that it measures adherence to physical therapy through a set of multiple choice questions that cover several domains known to affect adherence. It not only establishes a myriad of validities, but also indicates patients’ level of adherence in several categories based on cut-off values that are highly sensitive and specific. Furthermore, it highlights potential areas of improvement which could be addressed by healthcare professionals to empower patients in adhering to exercise as part of treatment. This would benefit patients and improve their participation subsequently improving their health-related quality of life.

The scale has potential to be used as a treatment outcome measure for chronic illnesses that affect the physical functioning of the body such as arthritic conditions. Moreover, it could be used to address factors that may act as barriers to treatment in acute illnesses that require rehabilitation for a longer period of time. For instance, poliomyelitis rehabilitation usually takes years to complete and it has been reported that financial issues and exhaustive treatment attendance may act as barriers [3, 18]. Such factors could be easily identified through GRAS and addressed earlier in therapy. Furthermore, the scale could be used in combination with other scales that measure quality of life, physical functioning and disability, to analyze how adherence to exercise impacts patient recovery.

Further to this, it was observed that educated patients were more adherent as compared to the uneducated ones. It is worth mentioning that educated patients make informed decisions about their health. Such patients have better health literacy and are able to understand their treatment needs. Thus, there is a likelihood that educated patients would know the importance of adhering to treatment [40].

Apart from its psychometric strengths, the GRAS could help therapists by highlighting the areas where patients lack in terms of adhering to PT. For instance, difficulty in managing time and prioritizing other commitments over PT sessions, are patients’ behavior related factors that could be measured by the scale. Based on the theory of planned behavior, patients’ belief towards physical therapy’s contribution in managing the illness would help in displaying a certain behavior, i.e., adherence [41]. If the issue is identified by the therapist, proper counseling and awareness regarding the importance of physical therapy in management of illness may modify patients’ attitude towards PT. If patient’s attitude is positive regarding importance of PT in illness, he/she may show an intent to behave in a certain way, i.e., prioritizing and attending PT sessions. This behavior (adherence) would help patient attain a positive health outcome, i.e., healthy state. This outcome would reinforce the positive attitude. Evidence highlights that behavioral interventions based on health belief model have created a positive perception about treatment in patients’ minds that have eventually resulted in better health outcomes [42].

Besides, the scale also measures non-adherence based on deliberate avoidance due to a feeling of wellness, as well as treatment resulted pain. This type of patient behavior could be explained with the help of the common-sense model of illness representation [43, 44]. Patient’s tendency to display a certain type of behavior is dependent on patient’s perception of health risk and associated emotional response to the risk [45]. If the perception about physical therapy is negative, such as that PT results in pain. The patient’s emotional response would be to avoid the therapy. If the patient is counseled regarding treatment induced pain in a way that dilutes the emotional response, i.e., avoidance to the risk (pain), patient may become less sensitive to PT resulted pain and together with a positive perception about treatment, may choose to undergo PT. Therefore, behavioral interventions such as patient counseling could help in modifying patients’ perception towards PT.

Moreover, the scale also identifies cost as a determinant of non-adherence. Evidence highlights that cost related non-adherence is common among patients in Pakistan [18, 38]. A qualitative study highlighted that patients in Pakistan may forgo treatment due to out-of-pocket expenditure [3]. The study further mentioned that physical therapists who provided discounts in consultation fee to patients helped increase patient retention and adherence [3]. However, discounted fee or financial assistance based on patients’ inability to pay, cannot be offered to every patient considering increasing healthcare costs borne by organizations as well as risk of compromising patient’s self-esteem. The GRAS tool could provide this detail to therapists. Lastly, unavailability of caregivers, i.e., accessibility issues and unavailability of human resource such as absence of female physical therapist may result in missing a PT session. These problems could be identified by the tool. Therapists could arrange a home visit for patients who have accessibility problems. It is not only beneficial for the patients but also for the clinics as it generates additional income. Female therapists could be arranged via networking among clinics.

Though, determinants apart from avoiding PT either due to wellness or pain, are of non-clinical nature, they affect a patient’s adherence to therapy session directly. The ease of identifying and addressing these issues through this tool makes it convenient to use in physical therapy clinics. This scale could be used at the time of patient’s history taking and a therapist could easily get an idea of patient’s adherence pattern at the beginning of treatment. Moreover, the therapist could spot areas for improvement and work with the patient. These aspects highlight the clinical utility of GRAS in daily practice.

Despite the notable strengths, our study had some limitations. The GRAS was developed in Urdu language since patients were of Pakistani origin. The English translation available in the manuscript has been validated however, the results are not published yet. Therefore, the publication of validation results of the English version of GRAS would be a pre-requisite to its international application. Secondly, the scale was validated holistically on patients undergoing physical therapy for musculoskeletal disorders. There may be differences in treatment outcomes for acute and chronic musculoskeletal conditions. Therefore, it is highly recommended to validate the scale in patients with specific musculoskeletal disorders.

## Conclusion

A novel scale to measure adherence to physical therapy was developed and validated. The tool has been validated using multiple approaches and incorporates several domains that affect a patient’s adherence to exercise in measuring adherence. The scale measures adherence to PT with high accuracy and provides scoring based on sensitive cut-off value which designates patients in different categories based on their level of adherence to exercise therapy. These aspects were absent in previously available scales. The availability of this tool would enhance monitoring for adherence in illnesses that require physical therapy either as an adjunct or sole treatment. It could also help clinicians and therapists address potential areas that may act as determinants of non-adherence. This would foster greater patient-therapist collaboration, improve satisfaction and increase patient participation.

## Supplementary information

**Additional file 1**. Urdu and English versions of General Rehabilitation Adherence Scale.

**Additional file 2.** The 8 – item General Rehabilitation Adherence Scale (GRAS) with scoring code.

**Additional file 3.** GRAS original 13 items with face and content validity results.

## Data Availability

The dataset generated are a property of the organization and is not publicly available. However, they are available from corresponding author on reasonable request.

## Abbreviations

CFI: Comparative fit index
CVI: Content validity index
CVR: Content validity ratio
EFA: Exploratory factor analysis
GRAS: General Rehabilitation Adherence Scale
ICC: Intra-class correlation
KMO: Kaiser-Mayer-Olkin
MLA: Maximum likelihood analysis
NFI: Normed fit index
PCA: Principle component analysis
PCFA: Partial confirmatory factor analysis
PKR: Pakistani Rupee
PT: Physical therapy
RMSEA: Root mean square error of approximation
SPSS: Statistical Package for Social Sciences
TLI: Tucker Lewis index
USA: United States of America
USD: United States Dollar
