# Supplementary material for: Development and validation of the General Rehabilitation Adherence Scale (GRAS) in patients attending physical therapy clinics for musculoskeletal disorders
Source: BMC Musculoskelet Disord. 2020 Feb 1;21:65. doi: 10.1186/s12891-020-3078-y (PMC6995046; doi:10.1186/s12891-020-3078-y)
Supplement: Supplementary file 1 — Additional file 1. Urdu and English versions of General Rehabilitation Adherence Scale. [file 12891_2020_3078_MOESM1_ESM.docx]

**The 8 – item General Rehabilitation Adherence Scale (GRAS – 8)**

**مریضوں کے باقاعدگی سے فزیوتھراپی کروانے کے عمل کو جانچنے کا آٹھ (8) نکاتی سوالنامہ**

| **Please choose the most appropriate answer for each of the following questions according to your physical therapy adherence** | | **درج ذیل سوالات کے لئے کسی ایک جواب کی نشاندہی کریں جو آپ کے فزیوتھراپی کروانے کی عادت کے مطابق ہو۔** |
| --- | --- | --- |
| **1.** | **Do you discontinue your physical therapy session because of other commitments?** | 1. **کیا ذاتی مصروفیات کے سبب فزیوتھراپی کروانا چھوڑ دیتے ہیں؟** |
| - Always - Mostly - Sometimes - Never | | - ہمیشہ - زیادہ تر - کبھی کبھی - کبھی نہیں |
| **2.** | **Do you discontinue your physical therapy session because you cannot manage time?** | 1. **کیا آپ فزیوتھراپی باقاعدگی/پابندی سے اس لئے نہیں کرواپاتے کیونکہ اس کے لئے وقت نکالنا مشکل ہوتا ہے؟** |
| - Always - Mostly - Sometimes - Never | | - ہمیشہ - زیادہ تر - کبھی کبھی - کبھی نہیں |
| **3.** | **Do you discontinue your physical therapy session when you feel well?** | 1. **کیا آپ جب بہتر محسوس کرتے ہیں تو فزیوتھراپی کروانا چھوڑ دیتے ہیں؟** |
| - Always - Mostly - Sometimes - Never | | - ہمیشہ - زیادہ تر - کبھی کبھی - کبھی نہیں |
| **4.** | **Do you discontinue your physical therapy session due to excessive pain caused by its intervention?** | 1. **کیا آپ جب فزیوتھراپی کے نتیجے میں درد محسوس کرتے ہیں تو فزیوتھراپی کروانا چھوڑ دیتے ہیں؟** |
| - Always - Mostly - Sometimes - Never | | - ہمیشہ - زیادہ تر - کبھی کبھی - کبھی نہیں |

| **5.** | **Do you discontinue your physical therapy session because you find it difficult to pay treatment cost?** | | 1. **کیا آپ فزیوتھراپی باقاعدگی/پابندی سے نہیں کرواپاتے کیونکہ اس کا خرچہ برداشت کرنا مشکل ہوتا ہے؟** |
| --- | --- | --- | --- |
| - Always - Mostly - Sometimes - Never | | | - ہمیشہ - زیادہ تر - کبھی کبھی - کبھی نہیں |
| **6.** | | **Do you discontinue your physical therapy session because it is not worth the amount of money that you had spent?** | 1. **کیا آپ فزیوتھراپی باقاعدگی/پابندی سے اس لئے نہیں کرواپاتے کیونکہ فزیوتھراپنی ادا کردہ ‌قیمت کے مطابق فائدہ مند نہیں ہے؟** |
| - Always - Mostly - Sometimes - Never | | | - ہمیشہ - زیادہ تر - کبھی کبھی - کبھی نہیں |
| **7.** | | **In the last month, did you skip your session when your caregiver (house driver, maid, nurse) was not available to accompany you to clinic?** | 1. **پچھلے مہینے میں کیا آپ نے اس وجہ سے فزیوتھراپی کا ناغہ کیا کہ آپ کے خیال کرنے والے مثلاً (گھر والے، میِڈ، ڈرائیور یا ہیلپر) آپ کے ساتھ کلینک جاتے وقت موجود نہیں ہوتے؟** |
| - Always - Mostly - Sometimes - Never | | | - ہمیشہ - زیادہ تر - کبھی کبھی - کبھی نہیں |
| **8.** | | **In the last month, did you skip your session when your physical therapist was not available?** | 1. **پچھلے مہینے میں کبھی آپ نے اس وجہ سے فزیوتھراپی کا ناغہ کیا کہ آپ کا فزیوتھراپسٹ موجود نہیں تھا؟** |
| - Always - Mostly - Sometimes - Never | | | - ہمیشہ - زیادہ تر - کبھی کبھی - کبھی نہیں |
