# Supplementary material for: Development and validation of the General Rehabilitation Adherence Scale (GRAS) in patients attending physical therapy clinics for musculoskeletal disorders
Source: BMC Musculoskelet Disord. 2020 Feb 1;21:65. doi: 10.1186/s12891-020-3078-y (PMC6995046; doi:10.1186/s12891-020-3078-y)
Supplement: Supplementary file 2 — Additional file 2. The 8 – item General Rehabilitation Adherence Scale (GRAS) with scoring code. [file 12891_2020_3078_MOESM2_ESM.docx]

**Additional file 2: The 8 – item General Rehabilitation Adherence Scale (GRAS – 8)**

| **S.No** | **Question** | **Categories** | **Grading** |
| --- | --- | --- | --- |
| 1. | Do you discontinue your physical therapy session because of other commitments? | Always  Mostly  Sometimes  Never | 0  1  2  3 |
| 2. | Do you discontinue your physical therapy session because you cannot manage time? | Always  Mostly  Sometimes  Never | 0  1  2  3 |
| 3. | Do you discontinue your physical therapy session when you feel well? | Always  Mostly  Sometimes  Never | 0  1  2  3 |
| 4. | Do you discontinue your physical therapy session due to excessive pain caused by its intervention? | Always  Mostly  Sometimes  Never | 0  1  2  3 |
| 5. | Do you discontinue your physical therapy session because you find it difficult to pay treatment cost? | Always  Mostly  Sometimes  Never | 0  1  2  3 |
| 6. | Do you discontinue your physical therapy session because it is not worth the amount of money that you had spent? | Always  Mostly  Sometimes  Never | 0  1  2  3 |
| 7. | In the last month, did you skip your session when your caregiver (house driver, maid, nurse) was not available to accompany you to clinic? | Always  Mostly  Sometimes  Never | 0  1  2  3 |
| 8. | In the last month, did you skip your session when your physical therapist was not available? | Always  Mostly  Sometimes  Never | 0  1  2  3 |
|  | **Grading for overall rehabilitation adherence (cumulative)**  High Adherence = 20 – 24 points  Good Adherence = 17 – 19 points  Partial Adherence = 12 – 16 points  Low Adherence = 8 – 11 points  Poor Adherence = 0 – 7 points |  |  |
