# Supplementary material for: Development and validation of the General Rehabilitation Adherence Scale (GRAS) in patients attending physical therapy clinics for musculoskeletal disorders
Source: BMC Musculoskelet Disord. 2020 Feb 1;21:65. doi: 10.1186/s12891-020-3078-y (PMC6995046; doi:10.1186/s12891-020-3078-y)
Supplement: Supplementary file 3 — Additional file 3. GRAS original 13 items with face and content validity results. [file 12891_2020_3078_MOESM3_ESM.docx]

**Additional file 3: GRAS original 13 items with face and content validity results**

|  | **Items** | **CVR** |
| --- | --- | --- |
| 1. | Do you discontinue your physical therapy session because of other commitments? | 0.80 |
| 2. | Do you discontinue your physical therapy session because you cannot manage time? | 0.84 |
| 3. | Do you discontinue your physical therapy session because or your job?* | 0.41 |
| 4. | Do you discontinue your physical therapy session because you have to stay home after work?* | 0.55 |
| 5. | Do you discontinue your physical therapy session when you feel well? | 0.94 |
| 6. | Do you discontinue your physical therapy session due to excessive pain?**  *Do you discontinue your physical therapy session due to excessive pain caused by its intervention?* | 0.87 |
| 7. | Do you discontinue your physical therapy session because you feel that it is not helping your condition?* | 0.33 |
| 8. | Do you discontinue your physical therapy session because it is expensive?* | 0.33 |
| 9. | Do you discontinue your physical therapy session because you find it difficult to pay treatment cost? | 0.99 |
| 10. | Do you discontinue your physical therapy session because it is not worth the amount of money that you had spent? | 0.8 |
| 11. | In the last month, did you skip your session due to unavailability of caregiver?**  *In the last month, did you skip your session when your caregiver (house driver, maid, nurse) was not available to accompany you to clinic?* | 0.99 |
| 12. | In the last month, did you skip your session due to unavailability of physical therapist?**  *In the last month, did you skip your session when your physical therapist was not available?* | 0.96 |
| 13. | In the last month, did you skip your session due to unavailability of your family?* | 0.33 |
| ** = removed, ** = modified* | | |
